# Mechanisms of Auditory Verbal Hallucination in Schizophrenia

**DOI:** 10.3389/fpsyt.2013.00155

**Published:** 2013-11-27

**Authors:** Raymond Cho, Wayne Wu

**Affiliations:** ^1^Center for Neural Basis of Cognition, University of Pittsburgh, Pittsburgh, PA, USA; ^2^Department of Psychiatry, University of Pittsburgh, Pittsburgh, PA, USA; ^3^Center for Neural Basis of Cognition, Carnegie Mellon University, Pittsburgh, PA, USA

**Keywords:** auditory hallucinations, schizophrenia, source monitoring, self-monitoring, spontaneous activation

## Abstract

Recent work on the mechanisms underlying auditory verbal hallucination (AVH) has been heavily informed by *self-monitoring accounts* that postulate defects in an internal monitoring mechanism as the basis of AVH. A more neglected alternative is an account focusing on defects in auditory processing, namely a *spontaneous activation account* of auditory activity underlying AVH. Science is often aided by putting theories in competition. Accordingly, a discussion that systematically contrasts the two models of AVH can generate sharper questions that will lead to new avenues of investigation. In this paper, we provide such a theoretical discussion of the two models, drawing strong contrasts between them. We identify a set of challenges for the self-monitoring account and argue that the spontaneous activation account has much in favor of it and should be the default account. Our theoretical overview leads to new questions and issues regarding the explanation of AVH as a subjective phenomenon and its neural basis. Accordingly, we suggest a set of experimental strategies to dissect the underlying mechanisms of AVH in light of the two competing models.

We shall contrast two proposed mechanisms of auditory verbal hallucinations (AVH): (a) the family of *self-monitoring* accounts and (b) a less discussed *spontaneous activity* account. On the former, a monitoring mechanism tracks whether internal episodes such as inner speech are self- or externally generated while on the latter, spontaneous auditory activity is the primary basis of AVH. In one sense, self-monitoring accounts emphasize “top-down” control mechanisms; spontaneous activity accounts emphasize “bottom-up” sensory mechanisms. The aim of this paper is not to provide a comprehensive literature review on AVH as there have been recent reviews (1, 2). Rather, we believe that it remains an open question what mechanisms underlie AVH in schizophrenia, and that by drawing clear contrasts between alternative models, we can identify experimental directions to explain what causes AVH. Self-monitoring accounts have provided much impetus to current theorizing about AVH, but one salient aspect of our discussion is to raise questions as to whether such accounts, as currently formulated, can adequately explain AVH. We believe that there are in fact significant limitations to the account that have largely gone unnoticed. Still, both models we consider might hold, and this requires further empirical investigation. Conceptual and logical analysis, however, will play an important role in aiding empirical work.

## Logical and Conceptual Issues Regarding Mechanisms of AVH

What is AVH? It is important to be rigorous in identifying what we are trying to explain, especially since clinical diagnosis of schizophrenia depends on patients’ reports of the phenomenology of their experience. Yet even among non-clinical populations, the concepts we use to categorize experiences may be quite fuzzy and imprecise (3). While there is little controversy that AVH involves language (emphasis on “verbal”) we restrict our attention to *auditory* experiences, namely where AVH is phenomenally like *hearing* a voice. Just as cognitive scientists distinguish between perception and thought in normal experience, we should where possible distinguish between auditory hallucination and thought phenomena (e.g., thought insertion). Admittedly, categorizing a patient’s experience as auditory or thought can be difficult, but we should first aim to explain the clear cases, those with clear auditory phenomenology. Finally, by “hallucination” we take a simple view: in AVH, these involve internal (auditory) representations that a verbalized sound occurs when there is no such sound.

**Figure 1:**
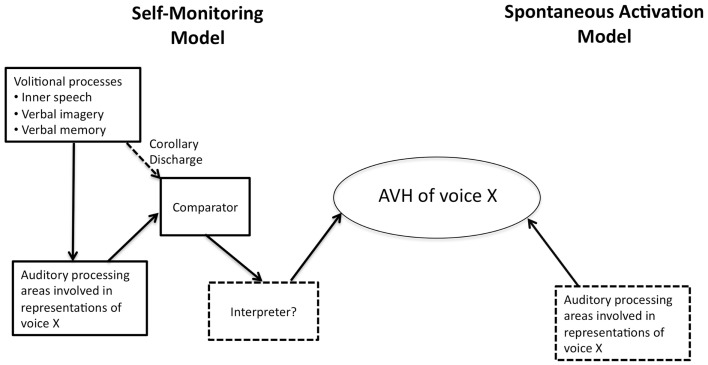
**Depiction of the two causal mechanisms for the generation of auditory verbal hallucination (AVH)**. The self-monitoring model is more complex than the spontaneous activity account.

One of the prevailing standard models of AVH invokes self- (or source-) monitoring (see Figure 1). This covers a family of models that share a core idea: a defect in a system whose role is to monitor internal episodes as self-generated. There is good evidence that patients with schizophrenia show defective self-monitoring on a variety of measures (4), and this may explain some positive symptoms such as delusions of control. It remains an open question, however, whether this defect is the causal basis of AVH.

We begin with proposals for the mental substrate of AVH: inner speech, auditory imagery, or auditory memory (5, 6). Most models take inner speech to be the substrate, but we think there are compelling phenomenological reasons against this (7, 8). Inner speech is generally in one’s own voice, is in the first-person point of view (“I”), and often lacks acoustical phenomenology (9). But even if there is controversy whether inner speech is auditory, there is no such controversy regarding AVH. As clinicians are aware, patients reflecting on the phenomenology of AVH typically report strong acoustical properties that are typically characteristic of hearing another person’s voice, where the voice typically speaks from a second- or third-personal point of view (“you” or “them” as opposed to “I”). Thus, what is typically represented in AVH experiences is starkly different from what is represented in normal inner speech experiences.

This point on phenomenological grounds indicates that a self-monitoring model that identifies inner speech as the substrate for AVH incurs an additional explanatory burden. It must provide a further mechanism that transforms the experience of the subject’s own inner voice, in the first-person and often lacking acoustical properties such as pitch, timbre, and intensity into the experience of someone else’s voice, in the second- or third-person, with acoustical properties. For example, how does inner speech in one’s own voice with its characteristic features become an AVH of, for example, the neighbor’s voice with its characteristic features? To point out that a patient mistakenly attributes the source of his own inner speech to the neighbor does not explain the phenomenological transformation. Indeed, once we allow that a given episode of AVH involves the features of another person’s voice with its characteristic acoustic features, it is simple to explain why the patient misattributes the event to another person: that is what it sounds like. Indeed, a phenomenological study found that in addition to being quite adept at differentiating AVH from everyday thoughts, patients found that the identification of the voices as another person’s voice was a critical perceptual feature that helped in differentiating the voices from thought (10). The point then is that inner speech models of AVH are more complex, requiring an additional mechanism to explain the distinct phenomenology of AVH vs. inner speech.

On grounds of simplicity, we suggest that self-monitoring accounts should endorse auditory *imagination* of another person’s voice as the substrate of AVH. If they do this, they obviate a need for the additional transformation step since the imagery substrate will already have the requisite phenomenal properties. Auditory imagination is characterized by acoustical phenomenology that is in many respects like hearing a voice: it represents another’s voice with its characteristic acoustical properties. Thus, our patient may auditorily imagine the neighbor’s voice saying certain negative things, and this leads to a hallucination when the subject loses track of this episode as self-generated. On this model, there is then no need for a mechanism that transforms inner speech phenomenology to AVH phenomenology. Only the failure of self-monitoring is required, a simpler mechanism. In this case, failure of self-monitoring might be causally sufficient for AVH. We shall take these imagination-based self-monitoring models as our stalking horse.

But what is the proposed “self-tagging” mechanism? We think self-monitoring theorists need more concrete mechanistic proposals here. The most detailed answer invokes corollary discharge, mostly in the context of forward models (11–13). This answer has the advantage that it goes beyond metaphors, and we have made much progress in understanding the neurobiology of the corollary discharge signal and forward modeling [see Ref. (14) for review]. These ideas have an ancestor in von Helmholtz’s explanation of position constancy in vision in the face of eye movements: why objects appear to remain stable in position even though our eyes are constantly moving. Position constancy is sometimes characterized as the visual system’s distinguishing between self- and other-induced retinal changes, and this is suggestive toward what goes wrong in AVH.

Forward models, and more generally predictive models, build on a corollary discharge signal and have been widely used in the motor control literature [see Ref. (15) for review; for relevant circuitry in primates controlling eye movement, see Ref. (16)]. The basic model has also been extended to schizophrenia (11–13), and a crucial idea is that the forward model makes a sensory prediction that can then suppress or cancel the sensory reafference. When the prediction is accurate, then sensory reafference is suppressed or canceled, and the system can be said to recognize that the episode was internally rather than externally generated.

There are, in fact, many details that must be filled in for this motor control model to explain AVH, but that is a job for the self-monitoring theorist. Rather, we think that failure of self-monitoring, understood via forward models, is still not sufficient for AVH, even with auditory imagination as the substrate. The reason is that there is nothing in the cancelation or suppression of reafference – essentially an error signal – that on its own says anything about whether the sensory input is self- or other-generated. Put simply: a zero error signal is not the same as a signal indicating *self* -generation, nor is a positive error signal the same as a signal indicating *other*-generation. After all, the computation of prediction error is used in other systems that have nothing to do with AVH, say movement control. Thus, while an error signal can be part of the basis of AVH, it is not on its own sufficient for it. Given the failure of sufficiency of self-monitoring models on their own to explain AVH, we believe that an additional mechanism beyond failure of self-monitoring will *always* be required to explain AVH. Specifically, the required mechanism, an interpreter, is one that explains the difference between self and other. The challenge for proponents of the account is to specify the nature of this additional mechanism [e.g., (17)]. As Figure 1 shows, the mechanism for AVH on self-monitoring accounts is quite complex.

We now turn to an alternative that provides a simple causally sufficient condition for AVH: AVH arise from spontaneous activity in auditory and related memory areas. We shall refer to this account as the *spontaneous activity account* [for similar proposals, see Ref. (2, 18, 19)]. The relevant substrates are the activation of specific auditory representation of voices, whether in imagination or memory recall (for ease of expression in what follows, we shall speak of activity in auditory areas to cover both sensory and relevant auditory memory regions, specifically the activation of representations of the voice that the subject typically experiences in AVH). We think that this should be the default account of AVH for the following reason: we know that appropriate stimulation of auditory and/or relevant memory areas is sufficient for AVH. Wilder Penfield provided such a proof of concept experiment.

Penfield and Perot (20) showed dramatically that stimulation along the temporal lobe resulted in quite complex auditory hallucinations. For instance, stimulations along the superior temporal gyrus elicited AVH with phenomenology typical of schizophrenia AVH, as noted in his case reports: “They sounded like a bunch of women talking together.” (622 p.); or more indistinct AVH – “Just like someone whispering, or something, in my left ear” or “a man’s voice, I could not understand what he said” (640 p.). Activity in these areas, spontaneously or induced, in the *absence* of an actual auditory input are cases of auditory hallucination: auditory experience of external sounds that do not in fact exist. The clinical entity epilepsy provides a similar, more naturalistic example of spontaneous cortical activity giving rise to AVH, in addition to a wide variety of other positive symptoms (21). The hypothesis of spontaneous activity is that AVH in schizophrenia derives from spontaneous auditory activation of auditory representations.

The basic idea of the spontaneous activity account is that the auditory system, broadly construed, encodes representations of previously heard voices and their acoustical properties. In the case of normal audition, some of these representations are also activated by actual voices in the environment, leading ultimately to auditory experiences that represent those voices. The idea of hallucination then is that these representations can be spontaneously activated in the absence of environmental sounds. Thus, the initiation of an episode of AVH is driven by spontaneous activation. Certainly, as in many cases of AVH, the experience can be temporally extended. This might result from some top-down influences such as the subject’s attention to the voice, which can further activate the relevant regions, leading to an extended hallucination. Indeed, there are reported cases of patients answering the AVH with *inner speech* in a dialog [12 of 29 patients in Ref. (9)]. Further, that top-down processes are abnormally engaged by bottom-up influences is suggested by the finding that there is a disturbance in connectivity *from* the superior temporal gyrus (sensory region) *to* the anterior cingulate cortex (involved in cognitive control) with AVH compared to patients without AVH and healthy controls (22). This raises two points: (1) that an inner speech response could induce additional activation of auditory representations and (2) that self-monitoring models that take inner speech as the AVH substrate are quite implausible here since the monitoring mechanism must go on and off precisely with the AVH and inner speech exchange.

Since the auditory representations spontaneously activated already encode a distinct person’s voice and its acoustical properties, there is no need for a system to interpret the voice represented as belonging to another person (recall the issue regarding inner speech above). That information about “otherness” is already encoded in the representation of another’s voice. In this way, the spontaneous activity account bypasses the complex machinery invoked in self-monitoring. On its face, then, the spontaneous activity account identifies a plausible sufficient causal condition for AVH that is much simpler than self-monitoring mechanisms. There are, of course, details to be filled in, but our point is to contrast two possible mechanisms: self-monitoring and spontaneous activity. We hope, at least, to have provided some initial reasons why the latter might be more compelling.

When a field is faced with two contrasting models, it must undertake specific experiments to see which may hold. Before we delve into concrete experimental proposals, we want to highlight some remaining conceptual points. First, the models are not contradictory, and thus both could be true. We might discover that across patients with AVH or across AVH episodes within a single patient, AVH divides between the two mechanisms. Second, both models make some *similar* predictions, namely that in AVH, we should see the absence of appropriate self-monitoring in an AVH episode, though for different reasons. On the self-monitoring account, this absence is due to a defect in the self-monitoring system; on the spontaneous activity account, this absence is due to spontaneous activity that bypasses self-monitoring. Critically, empirical evidence that demonstrates absence of appropriate self-monitoring during AVH does not support the self-monitoring account *as against the spontaneous activity account*. We need different experiments.

Finally, the spontaneous activity account is more parsimonious as seen in Figure 1, but is this really an advantage? Note that all accounts of AVH must explain (a) the *spontaneity* of AVH episodes (they often just happen) and (b) the *specificity* of phenomenology in AVH, namely negative content, second or third-person perspective, and a specific voice, identity and gender, etc. Both accounts can deal with the spontaneity: in self-monitoring, it is explained by spontaneous failure of self-monitoring so that AVH *feels* spontaneous; in the spontaneous activity account, it is the actual spontaneous activity of auditory areas.

A major challenge to all theories of AVH is to explain its specificity: why is it often a specific voice, negative in content, and focused on specific themes? Still, self-monitoring accounts face an additional challenge given that self-monitoring is a *general* mechanism applied to all internal episodes: why aren’t more internal auditory episodes experienced as “other.” For if a general self-monitoring mechanism for auditory processing fails, one would expect many more kinds of AVH: of their own voice as in playback, of environmental sounds, music, and so on [auditory non-verbal hallucinations are reported, but much less than voices; (23)]. Self-monitoring accounts could postulate a highly specific failure of self-monitoring, but note that this is no better than spontaneous activity theorists postulating spontaneous activation of specific representations. So, either self-monitoring mechanisms, being general, make predictions inconsistent with the facts or they are no better off on the specificity of AVH than the alternative spontaneous activation account.

## Potential Experimental Directions

In the previous section, we provided conceptual and logical grounds distinguishing two mechanisms of AVH and why we might prefer the spontaneous activity account. Still, the issues are fundamentally empirical. There are two plausible, starkly different mechanisms to explain AVH. What experiments might tease them apart? Studies in schizophrenia have characterized the neural correlates of the AVH-prone trait or actual AVH events, yielding important information about the potential functional and neuroanatomic basis for AVH, for instance, identifying areas involved in speech generation and perception [e.g., (24–26); also see meta-analyses by Jadri et al. (2); Modinos et al. (27); Palaniyappan et al. (28)], including evidence of competition for neurophysiologic resources that subserve normal processing of external speech (29). However, the studies have largely been correlative. For instance, the activity of Broca’s area as reported by McGuire et al. (24) could be interpreted as either due to sub-vocalizations that give rise to AVH *per se* but could also reflect responses to the AVH as commonly occurs. As such, prior work has largely lacked the experimental interventions that could establish causality and help to adjudicate between varying accounts. We outline potential interventions and their potential utility in deciding between the self-monitoring and spontaneous activity accounts.

The two accounts outlined above propose either lack of functioning (self-monitoring account) or inappropriate activation (spontaneous activation account) of neural/cognitive processes. Accordingly, potential experimental interventions could include perturbing self-monitoring processes vs. stimulation of sensory areas in healthy individuals to approximate the AVH experience reported by patients. One could also implement the same interventions in patients – perhaps to greater effect, as they could most authoritatively provide first-hand verification of whether such interventions reproduce AVH phenomenology. Finally, in patients who experience AVH as part of their illness, therapeutic interventions could remediate putative disturbances and could result in resolution of AVH symptoms; if such interventions could specifically target the processes under consideration, such an approach could provide strong support for a causal account of AVH (see Table 1).

**Table 1:** **Auditory verbal hallucination mechanisms: proof of concept studies**.

Intervention	Possible outcomes	Commentary
Disrupt self-monitoring during inner speech	AVH of one’s own inner voice (does not reproduce typical schizophrenia AVH)	To date, there have been no reports in the literature of such experiments for self-monitoring
Disrupt self-monitoring during auditory imagery	AVH of imagined voices	As above
Stimulate auditory cortices	AVH with typical phenomenology	There are relevant cases for stimulation of auditory cortices, e.g., neurosurgical studies (20); “naturalistic studies” [epilepsy; (21)]

### Testing self-monitoring accounts

Would perturbing self-monitoring processes provide evidence that it is the basis of AVH in schizophrenia? This is not clear. For example, a straightforward prediction of certain self-monitoring accounts that identify inner speech as the relevant substrate would be that perturbing self-monitoring processes in healthy individuals would be causally sufficient for the experience of hearing one’s own voice but with attribution to an external source. Concretely interpreted, it would be akin to listening to a playback of one’s recorded voice. This result, however, would only show that such perturbation yields a distinct form of AVH, namely hallucination of one’s voice as in a playback [though “replay” of patients’ thoughts or speech did not emerge as a significant contributor to the cluster analytic descriptions of the phenomenology; (19)]. Given the phenomenological differences from typical AVH, additional mechanisms are required to explain the full array of AVH characteristics, as noted in our “neighbor” example above.

A more plausible substrate for AVH is auditory imagination of another’s voice, so in principle, perturbation of self-monitoring during such imagination might yield AVH phenomenologically similar to that in schizophrenia. This seems a plausible test of self-monitoring accounts, though its implementation would require more concrete localization of the relevant self-monitoring mechanisms [Jadri et al. (2) noted midline cortical structures typically implicated in self-monitoring paradigms did not emerge as significant in their meta-analyses of imaging studies of AVH]. The relevant experiments are yet to be done, but the claim that failure of self-monitoring is sufficient for AVH raises the question, noted above: why don’t patients exhibit a wider range of AVH phenomenology?

What of interventions in patient populations vis-a-vis self-monitoring mechanisms? Employing a similar approach in schizophrenia patients would lead to similar predictions as in non-clinical populations, subject to the same logical constraints. Accordingly, upon experimentally interfering with self-monitoring during inner speech, a patient may report that they now have the experience of AVH of their own voice that is notably novel and distinct from their already existing AVH. Alternatively, with intervention during auditory imagination, they may report that their AVH has simply worsened in intensity/frequency, retaining similar phenomenology.

Nevertheless, there will always remain a logical gap between these inductions of AVH in both healthy and patient populations: the mechanisms of AVH could still be driven by spontaneous activation even though these interventions show that disruption of self-monitoring suffice for a form of AVH. Thus, what is required is to manipulate the putative mechanism during episodes of AVH in patients. This is not to say, however, that the previous experiments are useless. Far from it. Minimally, we can treat them as *proof of concept* experiments. They can demonstrate that such mechanisms can do the purported work, namely the induction of AVH. It is worth emphasizing a difference between the two mechanisms: while the spontaneous activation account already has a proof of concept result (e.g., Penfield’s work), no such proof has yet been done for self-monitoring in respect of AVH.

How then to directly manipulate the purported mechanism? Since the self-monitoring approach postulates a loss of function, an obvious manipulation is to see if AVH is ameliorated by restoration of this function. Cognitive behavioral therapies tailored to psychosis treatment have been successful at improving symptoms but the targets of therapy (e.g., distress from psychosis, depression) as well as the benefits (e.g., positive and negative symptoms) have been non-specific (30). While such approaches have clear clinical value, a more ideal approach for elucidating AVH mechanisms would entail interventions that are tailored to specifically target source monitoring processes. A case report of such an approach reported on a patient whose most prominent clinical symptom was daily thought insertion experiences and whose training involved improving their ability to accurately recall the source of self- vs. experimenter-generated items (31). Interestingly, the thought insertion symptoms did not improve as would be hypothesized, but an auditory hallucinations subscale showed improvement with training. So, while this finding is limited by interpretive issues, sample size, and a task that only indirectly taps self-monitoring processes (i.e., involving recall of sources rather than a more on-line measure), larger studies with refined task paradigms could yield a more definitive test of monitoring accounts of AVH. Conversely, given a means to safely perturb self-monitoring, following the logic for healthy individuals, one could similarly (further) impair this process in patients with similar considerations of the array of possible phenomenological outcomes. These experimental proposals are summarized in Table 2.

**Table 2:** **Auditory verbal hallucination mechanisms: testing self-monitoring account in schizophrenia**.

Intervention	Possible outcomes	Commentary
Disrupt self-monitoring	Worsening of existing AVH	As self-monitoring posits loss of function, it is unclear that disruption of self-monitoring during discrete AVH episodes provides relevant outcomes
	Increase in types of experienced AVH (e.g., inner speech)
		Further disruption of self-monitoring should lead to increase in AVH, but with predicted expansion of the type of AVH experienced (e.g., inner speech)
Remediation of monitoring deficits	Decrease in frequency of AVH	An ideal intervention of targeted remediation of self-monitoring during discrete AVH episodes in patients is currently unavailable
		General remediation studies have shown positive effects in AVH, but more studies are needed

### Testing spontaneous activity accounts

We have argued that a more parsimonious account of AVH involves susceptibility of relevant brain regions for spontaneous activation without external stimulation or volitional impetus. Such a mechanism could account for the full phenomenology of AVH, assuming that brain areas that represent the complex content and form of AVH show spontaneous activations of the sort found in normal auditory experience. Also consistent with the idea of spontaneous activation in sensory areas are findings from studies of auditory cortical responses in schizophrenia. The ability of cortical networks to coordinate their activity through gamma (30–80 Hz) oscillations is thought to be critical for the binding of perceptual features in creating coherent object representations (32). In studies of auditory processing, individuals with schizophrenia show reduced gamma synchrony compared with healthy controls (33–37). Interestingly, however, patients show correlations between auditory hallucination severity and gamma synchrony (38), consistent with the idea that a preserved excitability of sensory cortical areas is necessary for inappropriate spontaneous activations giving rise to AVH.

On this account, instabilities in sensory cortical areas would lead to spontaneous activations, but only do so in a coordinated fashion in those individuals with preserved ability to sustain gamma synchrony, thus giving rise to AVH. There would be an absence of AVH both in patients with reduced capacity for sustaining gamma synchrony as well as healthy individuals, due, respectively, to the inability to sustain such coordinated activity necessary for the perception of AVH or the lack of such instabilities that would inappropriately activate the cortex. Notably, such spontaneous activations could bypass any monitoring process since there is no inner speech or other self-initiated processes to issue a corollary discharge that would engage such monitoring, impaired, or otherwise.

The most definitive experiments to distinguish between the two models involve interventions in the activity of relevant auditory areas. The logic is as follows: the spontaneous activation account holds that the basis of AVH in patients with schizophrenia is the aberrant activation of relevant sensory areas. Accordingly, if it were possible to suppress such activation “on-line” while patients were experiencing AVH and show that such suppression, say by TMS, attenuated the hallucinatory experience, this would be good evidence that such activity was causally necessary for AVH in schizophrenia. An intervention to test causal sufficiency would be to then stimulate the areas identified as necessary to see if AVH of the same kind could be generated. Positive results along both dimensions would be strong support for the spontaneous activity account.

This proposal has been indirectly tested by Hoffman and other groups making similar use of TMS as a treatment for auditory hallucinations (39, 40). In contrast to the event-related design described above, the focus by Hoffman and others has been on clinical efficacy rather than elucidating of mechanisms. Accordingly, TMS interventions have been applied according to protocols whose timing is determined irrespective of their precise relationship to the specific episodes of AVH. While results are varied, meta-analyses confirm the utility of such a therapeutic approach (41, 42). So, while an “on-line” cortical suppression-mediated reduction in AVH would be more compelling, the general efficacy of such an approach is consistent with a local cortical spontaneous activation account.

One complication with the previous experiment arises *if self-monitoring accounts require the activity of relevant sensory areas in self-monitoring computations*. Thus, intervention in sensory areas might also affect the processes the self-monitoring account invokes to explain AVH. To make progress here, self-monitoring theorists must explain what computational role the sensory areas might play. There seem to us two possibilities. First, the sensory areas might compute the signal that is then compared against the corollary discharge signal so as to compute prediction error, as in forward modeling. If so, the experiment we have just proposed would help to adjudicate between the two models. On the spontaneous activity account, suppression of activation in sensory areas would suppress AVH. The opposite result would be seen in the proposed self-monitoring account. Self signals are associated with zero or low error when the corollary discharge is compared with the reafference signal, so when sensory areas are suppressed by TMS, the prediction is that computed error will be larger yielding an “other” signal. Thus, AVH should be exacerbated when activity in sensory areas is suppressed.

A second possibility is more congenial with self-monitoring accounts that invoke auditory imagery, since the imagery requires activation of relevant sensory areas (5, 43). On this view, the areas in question precisely are the substrate of the experience; what is critical is that the subject loses track of the fact that they are actively imagining. Consequently, AVH results. This version of self-monitoring would then agree with spontaneous activity accounts in predicting that suppression of activity in auditory areas during AVH yields reduced AVH. The difference between the models, then, is that the self-monitoring account takes sensory activation to be driven top-down. Accordingly, there is an additional way to disrupt AVH according to this version of self-monitoring: disrupting the top-down signal driving sensory activation. The spontaneous activity account holds that as there is no need for such top-down signals as the only way to manipulate AVH is via manipulation of the sensory area. To experimentally separate these models, we again need concrete mechanistic proposals from the self-monitoring account so that possible experimental manipulations can be designed (see Table 3).

**Table 3:** **Auditory verbal hallucination mechanisms: testing spontaneous activity account in schizophrenia**.

Intervention	Possible outcomes	Commentary
Inhibition of sensory cortex during discrete AVH episodes	AVH decrease	Demonstrates that a modulation of auditory cortical activity correspondingly modulates AVH severity
During quiescent non-AVH period, stimulation of same cortical region shown to be sensitive for inhibition of AVH	AVH with complete phenomenology	As above
Generalized inhibition of sensory cortex	Decrease in AVH frequency	Hoffman et al. (39, 40) have implemented generalized inhibition, demonstrating overall decrease in frequency of AVH
		A stronger test would be cortical inhibition during discrete AVH episodes as above

## Conclusion

There is much experimental work yet to be done on specific mechanisms for AVH. While we favor one model, our goal has been to clarify the conceptual landscape in the hopes of prompting more directed experiments to determine which is operative [though, as we noted, both or perhaps an integration of bottom-up and top-down accounts (44, 45) could be operative, yielding multiple mechanisms for AVH]. Having competing accounts on the field should aid focused inquiry on testing concrete mechanistic proposals. In doing this, we believe that we can make more progress toward understanding what causes AVH in patients with schizophrenia.

## Conflict of Interest Statement

The authors declare that the research was conducted in the absence of any commercial or financial relationships that could be construed as a potential conflict of interest.
